# Clinical use of vedolizumab subcutaneous formulation in inflammatory bowel diseases: a review of real-world evidence

**DOI:** 10.1007/s00384-025-04966-y

**Published:** 2025-08-16

**Authors:** Kirk B. Russ, Christian Agboton, Pravin Kamble, Abigail M. Wojtowicz, Anita Afzali

**Affiliations:** 1 Division of Gastroenterology and Hepatology, University of Alabama at Birmingham, Department of Medicine, 1808 7th Avenue South, Birmingham, AL 35233 USA; 2 Takeda, Cambridge, MA USA; 3 Division of Digestive Diseases, University of Cincinnati College of Medicine, Cincinnati, OH USA

**Keywords:** Ulcerative colitis, Crohn’s disease, Intravenous, Subcutaneous, Vedolizumab, Real-world evidence

## Abstract

**Introduction:**

Vedolizumab is an advanced therapy indicated for the treatment of moderately to severely active Crohn’s disease (CD) or ulcerative colitis (UC). After induction with vedolizumab intravenous (IV), maintenance is with vedolizumab 300 mg IV every 8 weeks, or patients can transition to vedolizumab 108 mg subcutaneous (SC) every 2 weeks.

**Methods:**

This was a literature review of the PubMed and Embase databases up to March 2024 to identify publications describing clinical practice experiences on the transition from vedolizumab IV to SC.

**Results:**

In total, 36 eligible publications were identified, comprising 4105 UC and CD patients treated with vedolizumab: 2718 with vedolizumab SC. Across studies, there was no loss of effectiveness after transition from vedolizumab IV to SC based on disease activity scores or biomarkers (C-reactive protein and fecal calprotectin). Higher vedolizumab trough levels were observed consistently with vedolizumab SC versus IV. Treatment persistence with vedolizumab SC ranged from 89.0% to 95.5% after 3 − 6 months and approximately 81% to 89% at 12 months. A willingness to transition to the SC formulation was reported by 47% to 59% of patients, due to time savings and fewer hospital visits. Patients who transitioned to vedolizumab SC showed a high level of satisfaction (75% − 95%) with the transition. The most frequently reported adverse events with vedolizumab SC were injection site reactions, with frequencies of 2.9% to 18.5%.

**Conclusions:**

Clinical practice experience in inflammatory bowel disease patients who transitioned from vedolizumab IV to SC showed no change in effectiveness or safety outcomes, and a high level of patient satisfaction overall.

**Supplementary Information:**

The online version contains supplementary material available at 10.1007/s00384-025-04966-y.

## Introduction

Crohn’s disease (CD) and ulcerative colitis (UC) are chronic relapsing and remitting inflammatory bowel diseases (IBDs) that require long-term treatment [1, 2]. Short-term treatment goals include improvement of symptoms and clinical remission, whereas longer-term treatment goals include endoscopic healing [3]. Vedolizumab is a gut-selective anti-α_4_β_7_ integrin therapy approved worldwide as an intravenous (IV) treatment for moderately to severely active CD and UC [4].

The preference for an IV or subcutaneous (SC) route of administration for maintenance therapy in IBD varies among patients. Whereas some patients prefer IV treatment to avoid self-injection, to reduce dosing frequency, or to increase contact with healthcare professionals, other patients may prefer an SC formulation because it is less time consuming and more convenient [5, 6]. Following development of the IV formulation for vedolizumab, an SC formulation of vedolizumab was developed to provide an alternative route of administration during maintenance treatment, enabling patients with UC and CD to choose their preferred route of administration for maintenance therapy [7]. Vedolizumab SC was evaluated in the phase 3 clinical trials VISIBLE 1 and VISIBLE 2 for the treatment of UC and CD, respectively [8, 9]. Overall, the efficacy and safety profiles of vedolizumab SC for maintenance therapy observed in the VISIBLE studies were consistent with those of the IV formulation observed in the GEMINI studies, with the exception of injection site reactions (ISRs) related to the difference in route of administration [10, 11].

The SC formulation of vedolizumab was approved in 2020 in Australia, Canada, and Europe as a maintenance therapy in adult patients with moderately to severely active CD and UC [12–14]; it was approved by the US Food and Drug Administration for UC in September 2023 and for CD in April 2024 [15].

The aim of this targeted literature review is to describe the effectiveness, safety, and treatment compliance of vedolizumab SC in patients with CD or UC in clinical practice under real-world conditions.

## Methods

In this targeted literature review, the PubMed and Embase databases were searched from January 1, 2019 up to 31 st March, 2024 to identify published journal articles and congress abstracts on the real-world use of vedolizumab SC. Boolean searches were performed using the following key search terms: “vedolizumab” AND “subcutaneous” AND “inflammatory bowel disease” OR “Crohn’s disease” OR “ulcerative colitis”; and the search was limited to observational studies in the English language. Abstracts of identified articles were screened by two independent reviewers for data on patients with IBD receiving vedolizumab SC in clinical practice. Publications were included if they reported data on the effectiveness, safety, treatment persistence, serum concentration, and patient satisfaction with the vedolizumab SC formulation, or data on patient willingness to transition from vedolizumab IV to the SC formulation.

Study information was extracted by the authors, duplicate studies were removed, and discrepancies were adjudicated. Data extracted from each study included number of patients, country, study setting and design, baseline characteristics, effectiveness based on disease activity scores (Harvey-Bradshaw Index, Simple Clinical Colitis Activity Index, Patient-reported outcomes, and partial Mayo score) and biomarkers (C-reactive protein and fecal calprotectin), safety (adverse events), treatment patterns (including treatment persistence), transition back to vedolizumab IV from SC, vedolizumab SC dose escalation, vedolizumab SC serum concentrations, patients’ satisfaction with vedolizumab SC treatment, and patients’ willingness to transition to vedolizumab SC treatment.

## Results

### Literature search

**Fig. 1:**
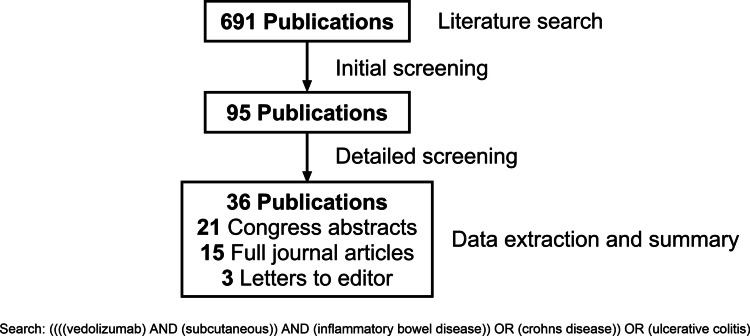
Literature search and screening

The initial literature search identified 691 publications (Fig. 1). After initial review, 95 publications were identified and screened in more detail; data were extracted from 36 eligible publications, including 21 congress abstracts [16–36] and 15 journal articles, 12 of which were full articles [37–48] and three of which were letters to the editor [49–51]. The 36 publications report data from studies carried out in Austria (*n* = 1), Belgium (*n* = 3), Croatia (*n* = 1), Denmark (*n* = 1), Finland (*n* = 1), France (*n* = 2), Germany (*n* = 4), Italy (*n* = 3), the Netherlands (*n* = 4), Norway (*n* = 1), Slovenia (*n* = 1), Spain (*n* = 3), Sweden (*n* = 2), Switzerland (*n* = 1), and the United Kingdom (*n* = 7).

### Patients

In total, 4105 patients were included from the publications, of whom 2718 patients with IBD (CD, 928; UC, 1350; IBD unspecified, 440) received vedolizumab SC in clinical practice. Following IV induction, vedolizumab SC can be administered as maintenance therapy at a dosage of 108 mg every 2 weeks [12, 15]. Patients can be transitioned at any time during maintenance from vedolizumab 300 mg IV every 8 weeks to the 108 mg SC formulation every 2 weeks, or they can be transitioned after the second IV induction dose. Nine publications included patients who started vedolizumab SC through switching from vedolizumab IV maintenance therapy [32, 34, 37–39, 41, 42, 46, 47]. Seven publications reported that some patients received vedolizumab SC maintenance immediately following IV induction [23, 24, 28, 36, 39, 45, 50]. Prior exposure to biologics at baseline varied among studies (Table 1) [17, 19, 23, 24, 34, 36, 41–44, 46, 47, 50].

**Table 1:** Key baseline characteristics in patients initiating VDZ SC therapy in clinical practice

	CD	UC	IBD unspecified
Proportion of patients with prior exposure to biologics	37%–100% [24, 36, 46, 50]	34%–100% [24, 36, 44, 46, 50]	45.8%–95% [17, 24, 39, 41–44]
Proportion of all patients receiving VDZ SC who switched during VDZ IV maintenance therapy	100% [27, 37, 41, 42, 46–48, 50]	60%–100% [27, 37, 41, 42, 46–48, 50]	39%–100% [28, 36, 38, 43, 45, 50]
Initial VDZ SC dose	108 mg q2w [37, 45, 48] Dose based on the prior VDZ IV dose [47]	108 mg q2w [37, 45, 48] Dose based on the prior VDZ IV dose [47]	108 mg q2w [39]

*CD* Crohn’s disease; *IBD* inflammatory bowel disease; *IV* intravenous; *q2w* every 2 weeks; *SC* subcutaneous; *UC* ulcerative colitis; *VDZ* vedolizumab

### Effectiveness

Seventeen publications reported effectiveness outcomes after transitioning from vedolizumab IV to SC in patients with IBD (Table 2) [18, 19, 24, 27, 31, 32, 34, 36, 37, 39, 41–43, 45–47, 49]. In the five publications that compared patients who chose to continue receiving vedolizumab IV maintenance with those who had transitioned to vedolizumab SC, there were no differences in effectiveness with respect to disease activity [27, 32, 34, 41, 45] or biochemical disease activity [34, 39, 41, 45] between the groups (Tables 2 and 3). In the seven publications that compared disease and biochemical activity from the time of transition to follow-up, there was no difference from the time of transitioning from vedolizumab IV to SC to the end of follow-up [34, 36, 37, 42, 46, 47, 49].

**Table 2:** Key effectiveness outcomes, treatment persistence, and dose escalation in patients with IBD receiving VDZ SC maintenance therapy in clinical practice

	CD	UC	IBD unspecified
Disease activity vs. baseline	Maintained (HBI, PRO2-CD) [36, 37, 45, 46] HBI decreased from 3.0 to 2.5 [31]	Maintained (PRO2-UC, pMayo, SCCAI) [31, 36, 37, 46] Significant reduction in SCCAI from 2 to 1.5 (*P* = 0.05) [45] Significant reduction in SCCAI (*P* < 0.05) [37]	
Proportion of patients in:			
Clinical remission	73%–82% [47]	88%–92% [47]	58%–100% [18, 27, 41, 42, 49]
CSFR	9.4%–68.2% [24, 46]	52.5%–65.2% [24, 46]	83% [39]
Biochemical remission	43.9%–66.7% [46]	55.0%–59.3% [46]	42%–89.4% [18, 19, 42, 49]
Combined CSFR and biochemical remission	NR	NR	89.2% [43]
Treatment persistence at: 3 months 6 months 12 months	NR 88.2% [41] 79.5% [41]	NR 89.4% [41] 82.3% [41]	92% [45] 89.0%–95.5% [37, 41, 45, 47] 81.2%–88.5% [34, 37, 41]
Proportion of patients with VDZ SC dose escalation	0% [45]–12.5% [37]^a^	0% [45]–7.3% [37]^b^	0% [45]–1.5% [46]

*CD* Crohn’s disease; *CSFR* corticosteroid-free remission; *HBI* Harvey–Bradshaw Index; *IBD* inflammatory bowel disease; *IV* intravenous; *NR* not reported; *pMayo* partial Mayo score; *NR* not reported; *PRO2* patient-reported outcome; *SC* subcutaneous; *SCCAI* Simple Clinical Colitis Activity Index; *UC* ulcerative colitis; *VDZ* vedolizumab

^a^One of these patients had received intensified IV dosing before transitioning to VDZ SC

^b^Two of these patients had received intensified IV dosing before transitioning to VDZ SC

**Table 3:** Change from baseline in key effectiveness measures, including biomarkers, for patients with IBD transitioning to vedolizumab subcutaneous

Parameter	Baseline value	Follow-up value
CRP	4 ± 0 mg/L [39]	5 ± 2 mg/L [39]
Calprotectin	259 ± 392 μg/L [39]	309 ± 367 μg/L[39]
IBD activity index scores	2 ± 3 [39]	2 ± 2 [39]

*CRP* C-reactive protein; *IBD* inflammatory bowel disease

### Treatment persistence

Treatment persistence was high, ranging from approximately 90% and above after 3–6 months of vedolizumab SC maintenance to approximately 80% at 12 months (Table 2) [34, 37, 41, 45, 47].

### Vedolizumab SC dose escalation

Two studies reported vedolizumab SC dose escalation (Table 2). In one, the vedolizumab SC dosing interval was reduced from 2 to 1.5 weeks in 1.5% of patients with IBD [46]. In the other, the dosing interval was reduced from 2 weeks to weekly in 10.1% of patients (12.5% CD and 7.3% UC), of whom three out of nine received an escalated dose of vedolizumab IV before transitioning [37]. A third study reported that no patients underwent dose escalation [45].

### Key safety outcomes and transition from vedolizumab SC back to IV

Six publications specified that ISRs were the most common adverse event in patients with IBD [34, 41, 43, 45, 47, 49], with rates ranging from 2.9% to 18.5% [34, 41, 45, 47]. The proportion of patients with IBD who transitioned from vedolizumab SC back to IV was 3.2% to 12.9% in 10 studies [25, 33–35, 40, 41, 43, 44, 46, 47] and 37.5% in one study [49] (Table 4 and Supplementary Fig. 1). Reasons for transitioning from vedolizumab SC back to IV included adverse events (including ISRs) [33, 41, 43, 46, 47], fear of needles [46], and patient preference [47]. The rates at which patients transitioned back to vedolizumab IV due to ISRs with SC administration were 2.8% to 6.5% (Supplementary Table 1) [41, 43, 47].

**Table 4:** Key safety outcomes in patients with IBD receiving VDZ SC maintenance therapy in clinical practice

	CD	UC	IBD unspecified
ISRs in patients receiving VDZ SC	NR	NR	Most common AE: ISRs [34, 41, 43, 45, 47, 49]
Proportion of patients who transitioned back from VDZ SC to IV	NR	6.0%[44]	3.2%–37.5 [25, 43, 49]
Outcomes after transitioning back from VDZ SC to IV	AEs: erythema at former SC injection site, urticaria, facial edema and swollen lips [50, 51]^a,b^	AEs: erythema at former SC injection site; headache, fatigue and abdominal pain; hypotension; urticaria; and cough, rhinorrhoea and dyspnea [50, 51]^a,b^	NR

*AE* adverse event; *CD* Crohn’s disease; *IBD* inflammatory bowel disease; *ISR* injection site reaction; *IV* intravenous; *NR* not reported; *SC* subcutaneous; *UC* ulcerative colitis; *VDZ* vedolizumab

^a^All of these patients transitioned back from VDZ SC to VDZ IV owing to AEs

^b^One publication only reported data from patients who transitioned back from VDZ SC to VDZ IV owing to AEs and went on to experience AEs during subsequent treatment

In the Volkers et al. cohort, of the four patients who transitioned from SC back to IV due to ISR, one patient had no further reactions; two reported mild allergic reactions (erythema), with one being successfully pre-treated with hydrocortisone and clemastine and the other requiring no pre-treatment; and one patient developed cough, rhinorrhea, and dyspnea despite clemastine pre-treatment, and discontinued vedolizumab IV [51].

In one publication that reported on 10 patients who transitioned from vedolizumab SC back to IV administration, seven patients experienced adverse events upon reverting to IV, six experienced allergic reactions, and one experienced hypotension. Four of these patients discontinued treatment [50].

### Willingness to transition and patient satisfaction with transitioning

Three publications reported on willingness to transition from IV to SC administration in patients with IBD (Table 5) [16, 20, 21]. One study reported on actual transition uptake among patients with IBD who were offered the opportunity to transition from IV to SC administration, with a rate of 59% [16]. Two studies reported on a hypothetical willingness to transition from IV to SC administration, with 47% to 56% of patients with IBD surveyed stating a hypothetical willingness to transition from IV to SC administration [20, 21].

**Table 5:** Patient perceptions of transitioning from vedolizumab IV to SC

Willingness to transition to vedolizumab SC	Patient satisfaction after transition from vedolizumab IV to SC
• 47%–59% of patients were willing to transition to vedolizumab SC [16, 20, 21] • Younger age was predictive of willingness to transition to vedolizumab SC [20] • Main drivers to transitioning were time-saving (70%–78%) and decrease in hospital visits (86%–90%) [16, 20, 21]	• There was a high degree of satisfaction with home administration, injection experience and the injector pen [22, 37, 45] • 75%–95% of patients were satisfied with the transition to vedolizumab SC [33, 49] • 80% of patients were happy with their decision to transition [38] • Patients found the SC devices easy to use [12]

*IV* intravenous; *SC* subcutaneous

One study found younger age to be an independent predictor of willingness to transition in a multivariate analysis [20] but another study found no significant predictors for willingness to transition relating to patient age, sex, diagnosis, drug, line of treatment (primary, secondary etc.), or duration of treatment [16, 38].

The main reasons for transitioning to vedolizumab SC were cited as “time saving” (70%–78%) and “decrease in hospital visits for infusion” (86%–90%) [16, 20, 21], whereas the main reasons to continue IV were “fear of change” (60%–61%) and “uncertainty in case of relapse” after transition to an SC formulation (46%) [20, 21].

The willingness to transition to vedolizumab SC was higher after a face-to-face approach (54%) compared with a digital approach (41%) [21]. For patients with doubts or hesitation about the transition, an electronic alert (71%), an information brochure (69%), and a personal teaching moment (60%) were recognized as valuable means of support [20, 21].

Seven publications reported that patients with IBD were satisfied with SC treatment after transitioning from IV treatment (75%–95%) [17, 22, 33, 37, 38, 45, 49].

### Vedolizumab serum concentrations following the transition from IV to SC administration

All nine publications that reported serum concentrations of vedolizumab after the transition from IV infusions to SC injection consistently showed an increase in serum concentrations [17, 18, 46, 47, 49] or serum trough concentrations of vedolizumab (Table 6) [37, 39, 42, 45]. This was a consistent finding for CD, UC, and all patients with IBD.

**Table 6:** Pharmacokinetics of transitioning from vedolizumab IV to SC

Vedolizumab serum concentrations after transition from IV to SC	CD	UC	IBD unspecified
Baseline	8.7–19 µg/mL [37, 46]	7.9–19 µg/mL [37, 46]	10–38.9 µg/mL [18, 39, 42, 45, 47, 49]
4 weeks	NR	NR	13.1 µg/mL [18]
6 weeks	NR	NR	40.7 µg/mL [47]
12 weeks	32 µg/mL [46]	31 µg/mL [46]	22.7–44.5 µg/mL [45, 47]
24 weeks	19–37 µg/mL [37, 46]	18.5–38 µg/mL [37, 46]	22.9–47.6 µg/mL [18, 42, 47]
1 year	NR	NR	40.77 µg/mL [49]

*CD* Crohn’s disease; *IBD* inflammatory bowel disease; *IV* intravenous; *NR* not reported; *SC* subcutaneous; *UC* ulcerative colitis

## Discussion

This review explored the clinical experience of the use of vedolizumab SC as maintenance treatment for patients with IBD. Real-world studies that compared patients with IBD who transitioned from vedolizumab IV to SC maintenance therapy with those who continued receiving IV maintenance therapy showed no difference in clinical effectiveness or safety, with the exception of ISRs.

The SC formulation of vedolizumab was studied in the clinical trials VISIBLE 1, VISIBLE 2, and VISIBLE open-label extension (OLE). These studies demonstrated that the efficacy of vedolizumab SC for maintaining clinical remission was significantly greater than placebo in patients with UC and CD, and was comparable to vedolizumab IV [8, 9]. These data are consistent with a meta-analysis of three real-world cohort studies that are also included in this review (Bergqvist et al., Volkers et al., and Wiken et al.) [37, 46, 47], in which a random-effects model showed no difference in the odds of maintaining clinical remission with vedolizumab SC versus IV in patients with UC or CD [52]. Overall, real-world evidence for effectiveness with vedolizumab SC in IBD is aligned with the outcomes demonstrated in the clinical trials. Treatment persistence with vedolizumab SC was high at approximately 90% and above after 3 to 6 months and approximately 80% at 12 months.

The VISIBLE studies also showed that the safety profile of vedolizumab SC was comparable to the IV formulation, with the exception of ISRs. Moreover, maintenance treatment with vedolizumab SC was well tolerated [8, 9]. Reported ISRs in the real-world evidence studies included in this review ranged from 4.1% to 18.5%, which is comparable to the rates of ISRs observed with vedolizumab SC during the VISIBLE 1 (10.4%) and VISIBLE 2 (2.9%) trials. In clinical trials, ISRs did not lead to discontinuation of, or changes to, the study medication dose, and the likelihood of experiencing an ISR trended down over time with increasing injection experience. In the real-world studies, some patients transitioned back to vedolizumab IV from the SC formulation due to ISRs. Some preliminary reports highlighted that patients who developed ISRs to vedolizumab SC who then transitioned back to IV administration are at risk of developing infusion reactions while receiving vedolizumab IV [50, 51]. Further studies with larger patient numbers are needed to confirm these findings and to assess the value of pre-treatment in preventing adverse events when transitioning back from vedolizumab SC to IV [51].

The studies that measured the serum concentration of vedolizumab after the transition to SC injection from IV infusions showed an increase in serum concentrations or trough concentrations of vedolizumab. A pooled analysis of data from four vedolizumab pivotal phase 3 trials has shown that exposure based on average serum concentrations for vedolizumab 300 mg IV every 8 weeks is equivalent to that for vedolizumab 108 mg SC every 2 weeks. This confirms that patient exposure to vedolizumab is comparable across both routes of administration. As expected for these two routes of administration, trough concentration for vedolizumab SC every 2 weeks is greater than that for vedolizumab IV every 8 weeks, whereas peak concentration for vedolizumab SC every 2 weeks is lower than that for vedolizumab IV every 8 weeks [53]. This concept is further demonstrated in a recent analysis by Wang et al. (2023) [54]. In addition, a recent study by Kolehmainen et al. showed that patients with UC who switched from maintenance therapy with vedolizumab IV to SC had significantly greater serum vedolizumab concentrations during vedolizumab SC treatment compared with vedolizumab IV therapy, while disease activity remained stable. In patients who switched to vedolizumab SC from vedolizumab IV induction therapy, serum vedolizumab concentrations remained unchanged, while clinical disease activity decreased significantly [55].

With the recent approval of vedolizumab SC, maintenance therapy with vedolizumab for the treatment of patients with IBD can now be self-administered. Self-administration of maintenance therapy via SC administration offers patients a convenient and time-saving option, as they do not have to travel to the hospital or infusion center facilities for IV infusions [51]. Furthermore, transitioning from IV to SC formulations allowed for reduced healthcare resource burden and reduced potential for viral transmission during the coronavirus pandemic [56].

Across the studies reviewed, the majority of patients surveyed were willing to transition to maintenance therapy with the vedolizumab SC formulation from IV [16, 20, 21]. For patients unwilling to transition, the reasons cited were “fear of change” and “concern regarding potential relapse” after transition to the SC formulation [16, 20, 21]. Other studies show some patients prefer an IV route of administration for the reassurance provided by the opportunity to interact with a healthcare professional or because they are averse to self-injection [5, 57, 58]. Similar reasons have been expressed by patients participating in a national survey in Israel, where the most common reasons for being unwilling to undertake a hypothetical transition from an IV to SC biologic formulation were a preference for interaction with a healthcare professional and a fear of needles [59]. Face-to-face education and support packages may be helpful in alleviating patients’ concerns regarding transitioning [20, 38, 60]. Among patients who did transition from vedolizumab IV to SC maintenance treatment, there was a high degree of satisfaction with the SC device, which was found to be easy to use and convenient, as patients are able to administer treatment at home. Overall, with the availability of both vedolizumab IV and SC, patients and physicians may choose the route of administration best suited for their needs.

Limitations associated with this work are that it is descriptive, with no statistical analyses of findings; therefore, any limitations in the source studies (e.g., lack of randomization, potential insufficient statistical power, use of non-validated questionnaires, or selection bias in the study populations) cannot be ruled out. Additionally, regional differences in healthcare systems, prescribing practices, or patient populations across different countries may influence the results. Nevertheless, this is the only consolidated source of available data on the real-world experience of the transition from vedolizumab IV to SC.

## Conclusion

With the recent approval of vedolizumab SC for patients with moderately to severely active UC and CD in the United States, we evaluated published data on its use in clinical practice in Europe and Canada. These data indicate that vedolizumab SC is a well-tolerated and effective treatment for the maintenance of clinical remission in patients with CD and UC treated in clinical practice, and treatment persistence with vedolizumab SC was high. Patients who showed a preference for the SC formulation noted its ease of use and convenience, and patient satisfaction with vedolizumab SC was high. Further well-designed, real-world studies, with long-term follow-up will add to our understanding of the experience of IBD patients who transition from vedolizumab IV to SC formulations.

## Supplementary Information

Below is the link to the electronic supplementary material.

Supplementary file1 (DOCX 265 KB)

## Data Availability

No datasets were generated or analysed during the current study.
