# Supplementary material for: Clinical use of vedolizumab subcutaneous formulation in inflammatory bowel diseases: a review of real-world evidence
Source: Int J Colorectal Dis. 2025 Aug 16;40(1):179. doi: 10.1007/s00384-025-04966-y (PMC12356757; doi:10.1007/s00384-025-04966-y)
Supplement: Supplementary file 1 [file 384_2025_4966_MOESM1_ESM.docx]

**Supplementary material**

**Supplementary Table 1** Summary of the Transition from Vedolizumab SC Back to Vedolizumab IV

| **Publication** | **Study type and Country** | **Patient population** | **Patient N** | **SC to IV Transition, n** | **Reasons for transition** | **Outcomes following SC to IV transition** |
| --- | --- | --- | --- | --- | --- | --- |
| Gros et al 2024 | Retrospective, observational, multicenter  Spain | UC, CD and IBD unspecified | 103 | 5 | Not reported | Not reported |
| Kubesch et al 2023 | Retrospective, observational  Germany | UC, CD and IBD unspecified | 82 | 5 | Local allergic reaction (n=1) | Not reported |
| Ribaldone et al 2024 | Prospective, observational, multicenter  Italy | UC | 168 | 10 | Not reported | 80% of patients recaptured clinical remission following switching back to VDZ IV |
| Bacsur et al 2023 | Prospective  Hungary | UC and CD | 24 | 9 | Not reported | Not reported |
| Lim et al 2023 | Retrospective, multicenter  United Kingdom | UC and CD | 410 | 39 | - Active disease (n=20) - Injection site reactions (n=12) - Minor systemic side effects: headache, fatigue, and nasopharyngitis (n=4) - Non-compliance or patient preference (n=3) | 33/39 returned to Q8W dosing and 6/39 at an escalated dose.  Of the 33 on Q8W dosing, 3 changed biologic therapy and 3 stopped biologic treatment. None changed to escalated VDZ IV dosing. |
| Parisio et al 2023 | Prospective, observational  Italy | UC and CD | 93 | 12 | Injection site reactions (n=6); disease flare (n=4); route of administration preference (n=2) | Not reported |
| Volkers et al 2022 | Prospective, multicenter  Netherlands | UC and CD | 135 | 12 | Unspecified AEs (n=9); fear of needles (n=3) | Not reported |
| Wiken et al 2023 | Prospective, single center  Norway | UC and CD | 108 | 5 | Injection site reactions (n=3); route of administration preference (n=2) | Not reported |
| Nørgaard et al 2021 | Prospective, non-interventional, observational  Denmark | UC and CD | 19 | 2 | Severe injection site swelling (n=1); leukopenia (n=1) | Not reported |
| Maw et al 2021 | Report of patients who switched to VDZ SC during the COVID-19 pandemic | UC and CD | 31 | 1 | Disease flare (n=1) | Not reported |
| Todeschini et al 2023 | Prospective  Italy | IBD unspecified | 76 | 8 | Unspecified AEs (n=8) | Not reported |
| Richard et al 2023 | Case report of 7 patients who switched back to VDZ IV who then experienced adverse reactions  France | UC and CD | 7 | 7 | Injection site reactions (n=7) | 7 patients experienced allergic reactions (urticaria n=3, erythema at former SC injection sites n=2, facial oedema n=1, hypotension n=1).  Allergic reactions led to VDZ discontinuation in 4 patients. |

**Supplementary Figure 1.** Proportions of Patients Who transitioned from Vedolizumab SC Back to Vedolizumab IV

**Supplement References**

Gros B, Manceñido Marcos N, Guardiola J, et al. P989 Switching from intravenous to subcutaneous vedolizumab in patients with inflammatory bowel disease in clinical remission: a multicenter study from GETECCU. J Crohns Colitis. 2024;18(Suppl 1):i1784–6. <https://doi.org/10.1093/ecco-jcc/jjad212.1119>.

Kubesch A, Kruse N, Jungheim F, et al. Switching Vedolizumab from IV to SC Injection in Inflammatory Bowel Disease Patients with Active Disease: Real-World Experience from a German IBD Cohort. J Clin Med. 2023;12(24). <https://doi.org/10.3390/jcm12247657>.

Ribaldone DG, Parisio L, Variola A, et al; Italian Group for the Study of Inflammatory Bowel Disease (IG-IBD). Switching from VEDOlizumab intravenous to subcutaneous formulation in ulcerative colitis patients in clinical remission: the SVEDO Study, an IG-IBD study. Dig Liver Dis. 2024;56(1):77–82. <https://doi.org/10.1016/j.dld.2023.07.011>.

Bacsur P, Iliás Á, Resál T, Kata D, Molnár T, Farkas K. Letter: Experience of switching vedolizumab treatment from intravenous to subcutaneous formulation in Hungary. Aliment Pharmacol Ther. 2023;58(2):262–3. <https://doi.org/10.1111/apt.17610>

Lim SH, Gros B, Sharma E, et al. Safety, effectiveness, and treatment persistence of subcutaneous vedolizumab in IBD: a multicenter study from the United Kingdom. Inflamm Bowel Dis. 2023;izad166. <https://doi.org/10.1093/ibd/izad166>.

Parisio L, Settanni CR, Varca S, et al. Effectiveness and Safety of Switching from Intravenous to Subcutaneous Vedolizumab Formulation in Inflammatory Bowel Disease Patients in Clinical Remission. J Gastrointestin Liver Dis. 2023;32(4):452-459. <https://doi.org/10.15403/jgld-5084>.

Volkers A, Straatmijer T, Duijvestein M, et al; IBD Center Amsterdam and the Dutch Initiative on Crohn and Colitis. Real-world experience of switching from intravenous to subcutaneous vedolizumab maintenance treatment for inflammatory bowel diseases. Aliment Pharmacol Ther. 2022;56(6):1044–54. <https://doi.org/10.1111/apt.17153>.

Wiken TH, Høivik ML, Buer L, et al. Switching from intravenous to subcutaneous vedolizumab maintenance treatment in patients with inflammatory bowel disease followed by therapeutic drug monitoring. Scand J Gastroenterol. 2023;58(8):863–73. <https://doi.org/10.1080/00365521.2023.2176252>.

Nørgaard J, Malte R, Charlotte P, et al. P602 Vedolizumab subcutaneous influencing disease course in Constant-care app solution J Crohns Colitis. 2021;15:S547-S548. <https://doi.org/10.1093/ecco-jcc/jjab076.723>

Maw F, Chan MY, Ritchie S, Fletcher V, Dallal H, Dhar A. PMO-27 Switching to subcutaneous vedolizumab in inflammatory bowel disease during COVID-19 pandemic – County Durham experience. Gut. 2021;70(Suppl 4):A91. <https://doi.org/10.1136/gutjnl-2021-BSG.166>.

Todeschini A, Geccherle A, Signoretto P, Fasoli E, Variola A. P422 Transition from intravenous to subcutaneous infliximab and vedolizumab: adherence, satisfaction and safety profile in a cohort of patients with inflammatory bowel disease. J Crohns Colitis. 2023;17(Suppl 1):i551–2. <https://doi.org/10.1093/ecco-jcc/jjac190.0552>.

Richard N, Vuitton L, Fumery M. Letter: Tricky reactions to switch back from subcutaneous to intravenous vedolizumab in patients with inflammatory bowel disease. Aliment Pharmacol Ther. 2023;57(6):741–2. <https://doi.org/10.1111/apt.17395>.
